# Adaptive Firefly Algorithm: Parameter Analysis and its Application

**DOI:** 10.1371/journal.pone.0112634

**Published:** 2014-11-14

**Authors:** Ngaam J. Cheung, Xue-Ming Ding, Hong-Bin Shen

**Affiliations:** 1 Institute of Image Processing and Pattern Recognition, Shanghai Jiao Tong University, and Key Laboratory of System Control and Information Processing, Ministry of Education of China, Shanghai, China; 2 School of Optical-Electrical and Computer Engineering, University of Shanghai for Science and Technology, Shanghai, China; Beijing University, China

## Abstract

As a nature-inspired search algorithm, firefly algorithm (FA) has several control parameters, which may have great effects on its performance. In this study, we investigate the parameter selection and adaptation strategies in a modified firefly algorithm — adaptive firefly algorithm (AdaFa). There are three strategies in AdaFa including (1) a distance-based light absorption coefficient; (2) a gray coefficient enhancing fireflies to share difference information from attractive ones efficiently; and (3) five different dynamic strategies for the randomization parameter. Promising selections of parameters in the strategies are analyzed to guarantee the efficient performance of AdaFa. AdaFa is validated over widely used benchmark functions, and the numerical experiments and statistical tests yield useful conclusions on the strategies and the parameter selections affecting the performance of AdaFa. When applied to the real-world problem — protein tertiary structure prediction, the results demonstrated improved variants can rebuild the tertiary structure with the average root mean square deviation less than 0.4Å and 1.5Å from the native constrains with noise free and 10% Gaussian white noise.

## Introduction

Firefly algorithm (FA) is a simple yet quite efficient nature-inspired search technique for global optimization. Since FA was developed, it has attracted a lot of attentions and becomes more popular in solving various real-world problems [1]–[6]. FA is a swarm-based intelligence algorithm, which mimics the flashing behavior of fireflies [7]. A firefly flashes as a signal to attract others for some purposes, e.g. predation or mating. Accordingly, this biological phenomenon is formulated as a meta-heuristic algorithm depending on following three rules [7], [8]:

- All fireflies are attracted by each other without respect to their sex;
- Attractiveness is proportional to its brightness, that is, the less bright one will move towards the brighter one;
- If there are no brighter fireflies than a particular firefly, it will move randomly in the space.

Similar to other heuristic algorithms, FA also has several control parameters to be tuned, for instance, the light absorption coefficient, the randomization control factor, and the population size, for good performance on different problems. The values of these control parameters greatly determine the qualities of the achieved solutions and the efficiency of the FA algorithm. Generally, it is a problem dependent task to select suitable control parameters for FA. As to a complex problem, it will be a hard case to deal with if the problem with numerous local optima, in which most optimization algorithms will be trapped [9]–[11]. Although it is highly important, there is no consistent methodology for determining the control parameters of a FA variant. Mostly, the parameters are fixed experientially or set arbitrarily within some predefined ranges.

In FA algorithm, there are two important issues: (1) the variation of light intensity; and (2) formulation of attractiveness [7]. However, these parameters are either set constants or fixed empirically in the traditional FA [7], which may make the algorithm inefficient for the problems with complex landscapes [3]. Researchers have made numerous contributions to the improvement of FA considering the alteration of the control parameters. For example, Gandomi et al. applied several chaos mechanisms to tune light absorption coefficient and attractiveness coefficient [12]. In ref. [1], a geometric progression reduction scheme for the randomization parameter was introduced in FA to enhance the solution quality. Coelho and Mariani adopted Gaussian distribution probability functions to tune the light absorption coefficient and the randomization parameter [3]. In ref. [2], a chaotic mapping operator and a chaotic component were used to generate initial solutions and replace the random component of the standard FA, respectively. The former was to improve the quality of the initial population, while the latter was to perform ergodic search of the solution space.

Although much progress has been achieved on the FA-based algorithms since 2008, more efforts are required to further improve their performance:

- Providing the sufficient analysis for the control parameter settings;
- Efficient strategies or mechanisms for the selections of the control parameters;
- Employing heterogeneous search rules to enhance the performance of FA.

In this paper, the contributions are to develop several mechanisms and strategies for improving standard firefly algorithm, involving: (1) distance-based light absorption coefficient and gray coefficient for adaptively altering the attractiveness and enhancing difference information sharing, respectively; (2) five strategies for controlling the randomization parameter; and (3) employing heterogeneous search rules supported by the adaptively altering control parameters, to enhance the search ability of the original FA. The control parameters are adjusted over time or depending on heuristic rules, which employ the evolutionary information among the fireflies.

## Methods

### Firefly algorithm

The firefly algorithm (FA) is a nature-inspired optimization method [7], which maintains a population of fireflies to find the global optimum of an optimization problem.

In FA, the distance between any two fireflies 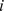 and 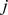 at 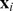 and 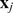, respectively, can be defined as the Euclidean distance 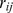, which is formulated as follows,
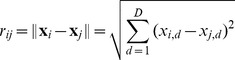
(1)where 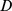 is the dimension of an optimization problem.

Indeed, the larger the distance 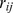 is, the less light the fireflies can see from each other. Accordingly, it is necessary to define monotonically decreasing functions for light intensity and attractiveness, respectively. They are presented in Eqs. (2) and (3).
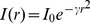
(2)where 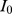 is initial light intensity, and 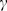 is the light absorption coefficient, which controls the decrease of light intensity. Accordingly, the attractiveness 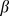 of a firefly is defined as shown in Eq. (3).
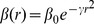
(3)where 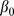 is a constant, which is the attractiveness at 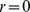.

The step of a firefly 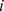 is attracted to move to another more attractive (brighter) firefly 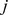 is determined by
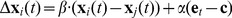
(4)where 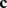 is a constant vector 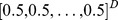 and 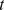 is the time step, 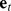 is drawn from a normal distribution 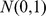. 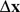 is the step size of the 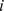
th firefly moving. The first term is the attraction from the 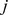
th firefly, while the second term is randomization controlled by 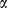, which is a constant in the range of 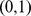. Therefore, the update of the 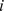
th firefly is formulated as follows,
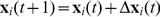
(5)


The Eqs. (4) and (5) show that the 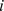
th firefly will move towards the 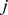
th firefly, which is a more attractive one.

The procedure of FA algorithm is summarized as follows (*Algorithm S1*):

The selections of parameters are crucially important in FA, such as the light absorption coefficient 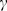 in Eq. (3) and the randomization parameter 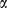 in Eq. (4), but the values of the control parameters are chosen in predefined ranges dogmatically [13]. During the search process, traditional FA does not alter the values of the control parameters or only use constant parameters throughout the whole process. Also the information of the search or the knowledge achieved by the fireflies are not taken into account in the selections of parameters. All these static designs may be optimal for one problem, but not efficient or even fail to guarantee convergence for another one [13]. Proper selections of these parameters highly determine the quality of the solution and the search efficiency. Although researchers have proposed many improved FA variants [1], [14]–[16], premature convergence can still occur in the original firefly algorithm and its variants. FA may be easily trapped in local regions when it is used to deal with the complex problems with numerous local optima if the randomness is reduced to quickly [3]. To overcome these weaknesses in FA, we develop five variants of FA based on two main mechanisms, which will be described in details in following sections.

### Adaptive firefly algorithm

The standard FA employs three parameters for solving the optimizations, and the parameters may result in significantly different performance of FA, such as the absorption coefficient 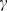 and the randomization parameter 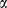. Proper selections of these parameters can be a useful way to improve the search ability of FA. However, considering different problems with distinguish features, it is difficult to manually tune the parameters. To enhance FA, two main mechanisms and five strategies are employed to avoid the premature convergence of the classical FA. Accordingly, five variants of FA are yielded to balance the exploitation (local search) and exploration (global search), which are denoted as AdaFa-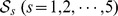. The two main mechanisms are distance-based adaptive mechanism for different information sharing and gray-based coefficients for efficiently enhancing the heterogeneous search. All the two mechanisms are adaptive to exchange messages and applied to tune the control parameters in FA. Additionally, we also propose five different strategies for the selection of the randomization parameter 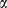 in Eq. (4).

#### Distance-based adaptive strategy

The motivation for us to investigate a distance-based strategy is that the adaptive absorption coefficient can efficiently deal with different problems whatever their landscape are, while the traditional FA uses a constant one throughout the search process.

It is obvious that there are two limited cases for a constant light absorption coefficient 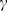 [4], which can be concluded as follows:

- The attractiveness of other fireflies will be a constant when 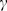 approaches 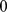. That is, a firefly can be seen by all the other ones. In this case, FA is the same as a classical PSO.
- If 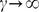, the attractiveness will be equal to 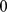. All the fireflies cannot take their bearings to move but in random flight. In this case, FA becomes a pure random search algorithm.

As can be seen, the parameter 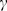 is crucially important in characterizing the variations of the attractiveness, and the speed of the convergence is also dependent on 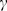 [12]. As a result, the performance of FA will be significantly constrained when a constant 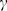 is used to solve the optimization problems as done in traditional FA. As is well known, the attractiveness should be linked with the distance among the fireflies, and it should also vary with the different distances among the population during the search process. The information of the distances is useful for promising search adaptively. Hence, we propose to use the distance to adaptively adjust the trajectories of the fireflies. The mean distance of the 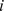
th firefly to the other fireflies is calculated as follows,
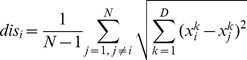
(6)where 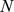 is the number of the fireflies.

Based on the distance calculated in Eq. (6), we then define the distance ratio as follows,
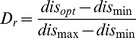
(7)where 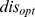 is the distance from the 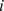
th firefly to the global best firefly. 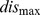 and 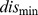 are the maximum and the minimum distances from the 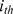 firefly to other fireflies, respectively.

**Figure 1:**
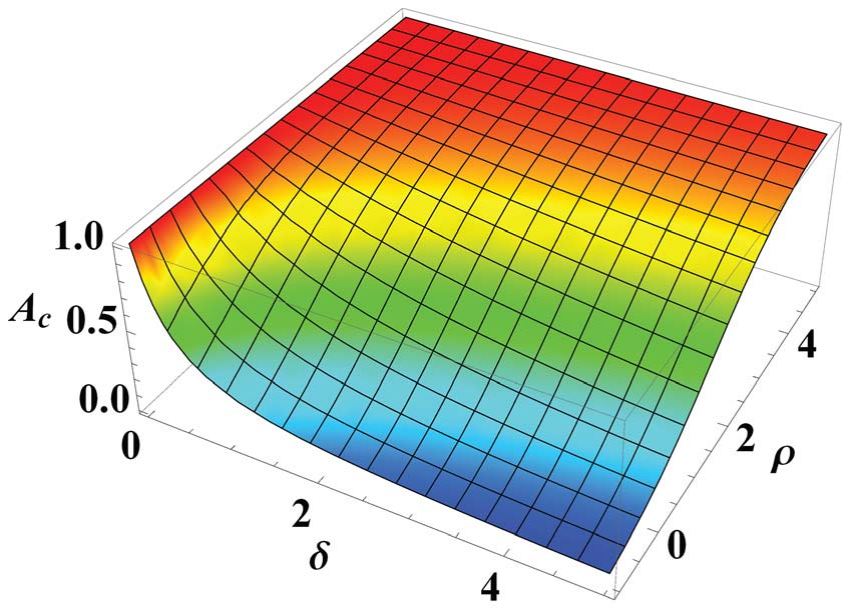
The relationship among 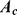, 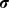 and 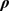. As 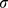 and 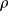 increase, 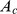 will decrease while the value of 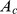 will sharply increase when 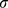 and 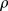 are very small (or large).

Accordingly, we define an adaptive absorption coefficient based on the distance ratio, which is to adaptively track the promising flight direction. It is defined as follows,
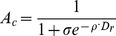
(8)where 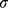 denotes as amplitude factor, which controls the amplitude of 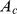. 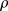 is called contraction index. The relationship between 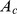 and the two factors is illustrated in Fig. 1.

In Fig. 1, the value of 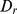 is set to 1, which does not have effect on the lower and upper boundaries of 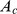. As can be seen, the larger the values of 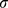 and 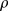 are, the smaller the value of 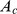 is; vice versa. If we remove the effect of 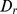, that is 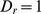, then 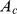 will be a constant. As a result, it is greatly difficult to deal with the balance between exploration and exploitation. Because the light absorption coefficient is determined by the distance information adaptively, the fireflies are able to adjust their flight directions for promising search.

Then, the constant light absorption coefficient 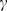 in traditional FA is replaced by the adaptive coefficient 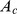 for efficient search. The new attractiveness is obtained in Eq. (9).
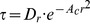
(9)


#### Gray-based coefficients

Gray relational analysis (GRA) is a similarity measure for finite sequences with incomplete information [17]. Let 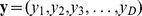 be the reference sequence, and 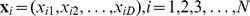 be the 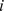
th comparative sequence, then the gray relational coefficient between 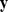 and 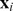 can be defined as
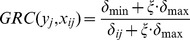
(10)where 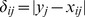, 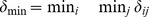, 
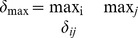
, and 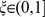 is employed to distinguish 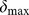 from 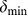 and called as distinguishing constant. Based on Eq. (10), we can calculate the corresponding gray relational grade as follows
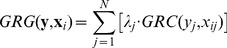
(11)where 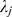 is a weighted constant of the gray relational coefficient 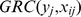 and subjects to 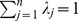.

To properly use the gray relational analysis, the new best firefly 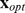 is employed as the reference sequence, while all the fireflies are the comparative ones. Base on these assumptions, we can use the gray relational analysis to measure the similarity between them. Let the gray relational grade between the new best firefly 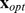 and the 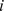
th firefly 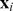 be 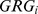. As can be seen from Eq. (11), the closer the new best firefly 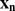 and the 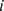
th firefly are, the larger the 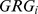 is. Accordingly, the relational grade 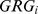 can be used to control the diversity of the firefly population. Since the gray relational grade involves the information of population distribution, we define a gray coefficient 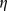 to satisfy the requirement of diversity as follows,
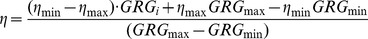
(12)where 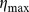 and 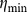 are the upper and the lower boundaries, which ensure the population can converge in finite time. 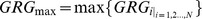 and 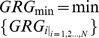.

#### Update rules

Fireflies in the traditional FA and most of its modifications, follow the same search law and share similar information throughout the search process. Due to the same search characteristics, the fireflies cannot always exhibit diverse and useful information for promising search. In an optimization algorithm, it is important to balance the exploration and exploitation. The algorithm should make contribution to exploration initially, while focusing on exploitation and convergence in the later search process [3], [18]. To accomplish the task, we introduce heterogeneous update rules to improve the search abilities of the fireflies. Two updating equations are defined and selected randomly in the search process, which are presented in Eq. (13).
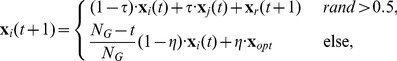
(13)where 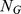 is the maximum number of generations. 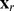 is the randomization term, which is defined based on new 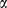-strategies as follows,
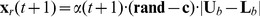
(14)where 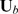 and 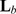 are the upper and lower boundaries, respectively.

In FA, the strategy (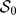) for 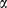 is
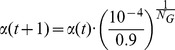
(15)


As can be seen from Eq. (15), 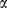 decreases linearly depending on the generation number, and it does not always work well for different problems. As an important parameter controlling the randomization step, 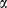 has significant effect on the performance of FA. To enhance its search ability, we propose five different strategies for 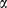, which all decrease dynamically with the generation number, population size and the size of the optimized problem. These designs promise AdaFa to deal with different types of problems, and all of them increase the range of 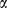. The strategies are presented in Eq. (16)–Eq. (20).

- strategy 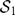

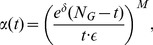
(16)where 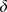 is constant defined by user to clamp 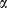, and 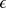 is also a constant, which is 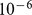.

- strategy 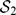


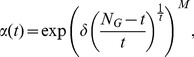
(17)•strategy 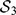

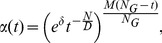
(18)where 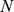 and 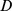 are the population size and the size of an optimization problem, respectively.

- strategy 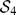

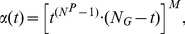
(19)where 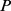 is the power of population size 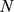.

- strategy 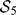

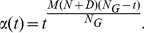
(20)In Eq. (16)–Eq. (20), 
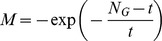
. From Eq. (16)–Eq. (20), 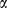 varies with time 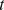 non-linearly, and the analysis of the parameters in the strategies are presented in following section.

Based on the designs of different adaptive mechanisms and strategies discussed above, the fireflies are allowed to learn more useful information from others and adjust the flight directions adaptively. The AdaFa algorithm is summarized in (*Algorithm S2*).

## Results

In this section, we demonstrate the performance of AdaFa variants over twelve benchmark functions 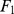
–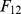 summarized in Table S1 of Supplementary Materials (more details can be referred to the study [19], [20]) and apply AdaFa variants to rebuild protein tertiary structure. Firstly, we conduct numerical experiments for parameters analysis and then compare AdaFa with standard particle swarm optimization (SPSO) [21], adaptive particle swarm optimization (APSO) [19], grey particle swarm optimization (GPSO) [20] and FA [8]. In numerical experiments, we analyze the parameter sensitivity, provide the parameter settings of each algorithm, present the results of the numerical experiment, and analyze the numerical results.

### Parameter sensitivity analysis

In this section, we analyze four parameters in AdaFa including the amplitude factor 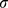, the contraction index 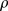, 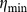 and 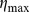. The different selections of the four parameters may have influence on its performance. To investigate the impacts of these parameters, we conducted the experiments on functions 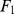
–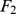, 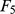, and 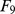
–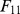, which are Sphere, Schwefel, Rosenbrock, Rastrigin, Ackley and Griewank functions, respectively. The maximum number of generations was set to 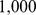, and the population size was 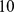 (This is similar to ref. [8], where the authors analyzed the effect of population size on the optimization problems). The averages of best-so-far values were used to measure the performance of AdaFa.

**Figure 2:**
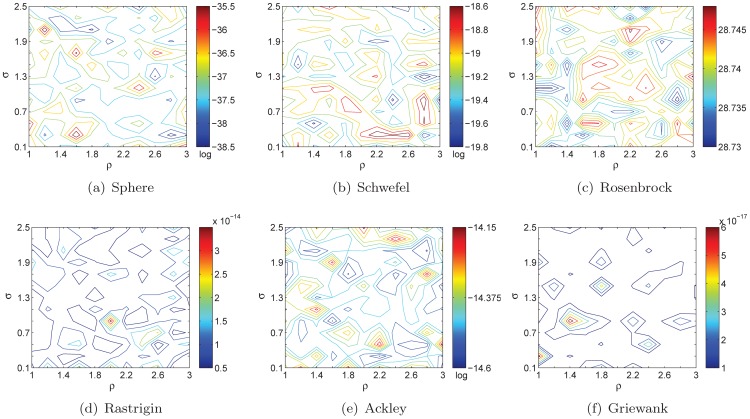
The relationship between the two factors (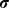 and 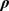) and the performance of AdaFa.

To investigate the amplitude factor 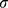 and the contraction index 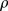, 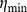 and 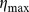 were set to 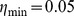 and 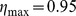, respectively. AdaFa was performed 20 times with different 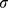 and 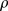. 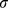 was varied from 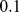 to 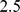 with an increment of 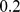, while 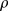 was changed from 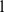 to 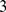 in the same increment. Fig. 2 reveals that the relationship between the best-so-far and the two factors. As can be seen, the optimal 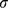 and 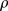 were around 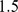 and 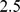, respectively. Hence, in this study 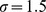 and 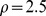 were used for all the following experiments.

**Figure 3:**
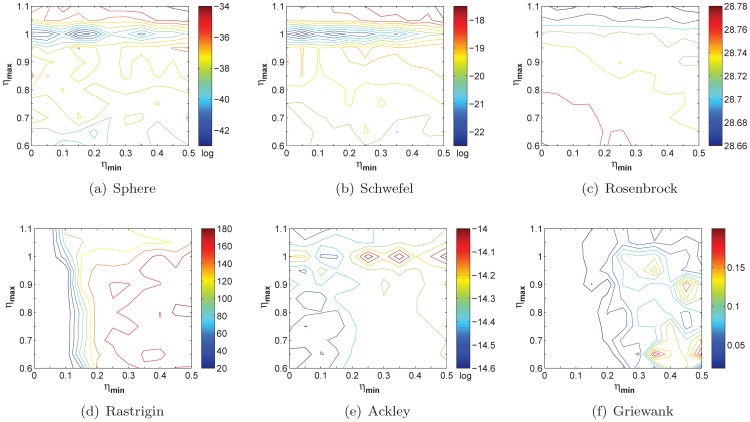
The relationship between 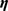 and performance of AdaFa.

In the gray strategy, we employ a gray coefficient 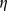 to trade-off between the 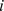
th firefly and the best firefly. As shown in Eq. (12), it is necessary to clamp 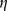 in a fixed interval, such as 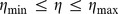. To fix 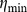 and 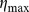, we conducted trial tests on the same six benchmark functions, and the maximum number of generations was also set to 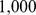. 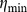 and 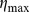 were set in 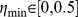 and 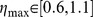, respectively, with the same increment of 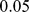. Fig. 3 shows the relationship between 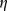 and performance of AdaFa. From Fig. 3, AdaFa will perform well when 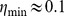 and 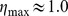. Hence, in this study, we use 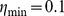 and 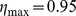 to conduct the experiments.

**Figure 4:**
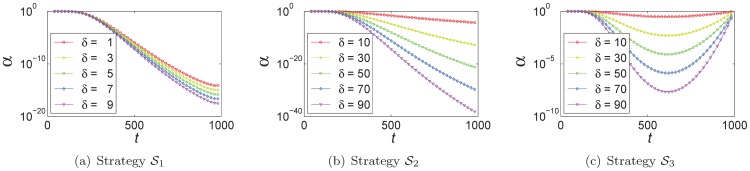
Different strategies for 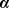: (a) strategy 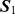, (b) strategy 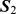, (c) strategy 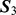 varying with different 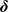.

In strategies 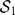 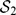 and 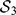 of 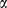 (Eq. (16)–Eq. (18)), 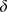 can be defined by user before the optimization. How to select 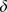 is important to 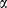. We thus analyze the relationship between 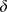 and 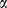, which is illustrated in Fig. 4. As shown in Fig. 4, the larger 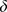 is, the larger the range of 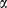 is. It is interesting to note that there is a tail in strategy 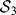 at the latter of generations, which can enhance the search abilities of the fireflies in exploitation region at latter search process.

**Figure 5:**
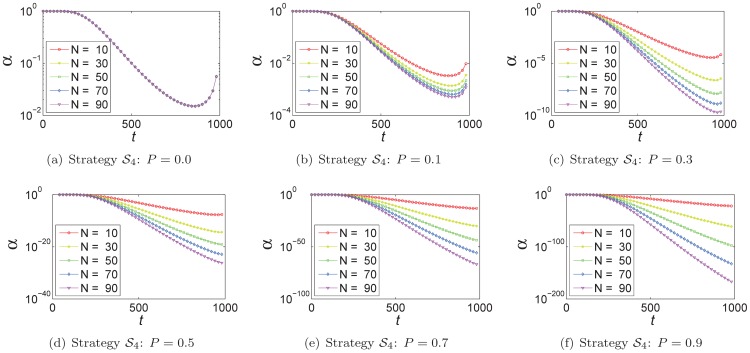
In strategy 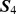, the relationship between 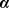 and the population size 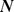 varying with different power value 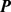.

**Figure 6:**
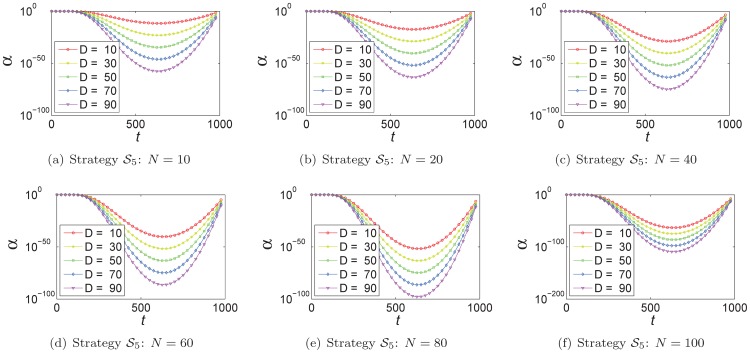
In strategy 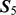, the relationship between 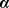 and the size of problem 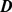 with different population size 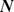.

In strategy 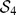 and strategy 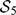, the values of 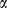 are dependent on the population size 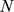 and the size 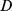 of an optimization problem, and we also analyze the relationship among them. As illustrated in Fig. 5, in strategy 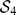 the smaller 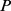 is, the more similar the trajectories of 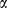 are. As well as strategy 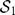
–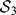, the range of 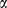 is enlarged with the increment of the value of 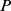 and population size 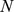. In strategy 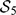, as shown in Fig. 6, although the trajectories of 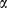 are different from each other varying with 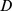, they all converge to similar points. These points are independent of the population size 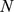.

**Figure 7:**
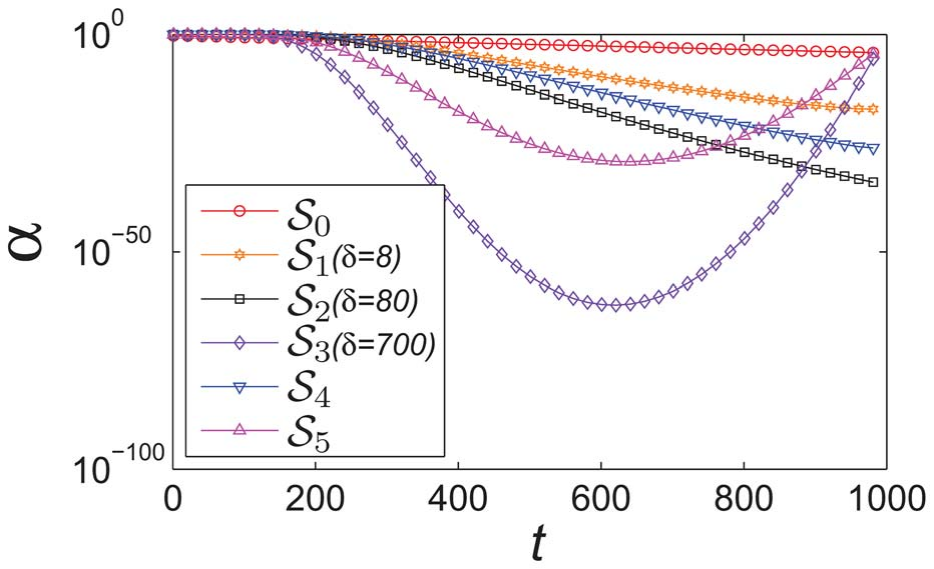
The comparison of different strategies for the randomization parameter 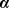.

In Fig. 7, different strategies of 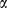 are compared, where the ranges of 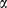 of all proposed strategies are larger than the strategy in standard FA. The larger ranges allow the fireflies with stronger exploration and exploitation abilities throughout the search process.

### Parameters settings

Experiments were conducted to compare different algorithms on 12 benchmark functions with 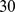 dimensions. In these experiments, the maximum number of generation was set to 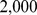, and the population of each algorithm was set to 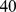. According to previous analysis, the parameter settings are listed as follows:

- SPSO [21]: 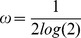, 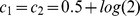.
- APSO [19]: 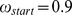, 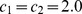.
- GPSO [20]: 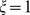, 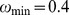, 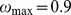, 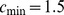, 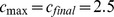

- FA [8]: 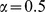, 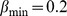, 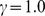.
- IFA: 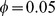, 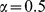, 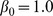, 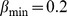, 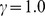.
- AdaFa-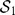: 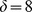, 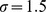, 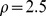, 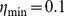, 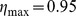.
- AdaFa-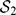: 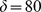, 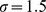, 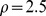, 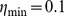, 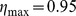.
- AdaFa-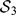: 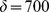, 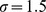, 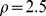, 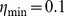, 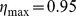.
- AdaFa-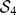: 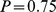, 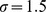, 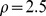, 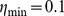, 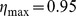.
- AdaFa-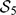: 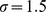, 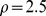, 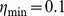, 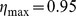.

### Numerical Results

The experimental results over the twelve benchmark functions are presented in this section. Table 1 shows the mean values and the standard deviation of the results over 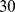 independent trials of each algorithm, and the success rate. The best are highlighted in bold, and the mean values are also illustrated in Fig. S1 in File S1 to compare the convergence of each algorithm (more details can be found in Supplementary Materials).

**Table 1:** The results achieved by different algorithms on the benchmark functions.

Algorithm	Function											
	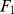			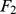			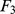			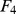		
SPSO	**0.00E+00**	±	**0.00E+00**	1.54E+00	±	8.56E-01	3.55E-04	±	3.68E-04	1.98E+00	±	1.36E+00
APSO	2.47E-37	±	7.47E-37	3.02E-20	±	1.56E-19	5.88E+00	±	7.02E+00	6.62E-01	±	3.30E-01
GPSO	2.83E-23	±	8.63E-23	6.15E-10	±	1.65E-09	2.15E+01	±	1.67E+01	1.20E+00	±	7.70E-01
FA	1.22E-03	±	2.53E-04	4.80E-02	±	3.53E-02	3.67E+01	±	2.83E+01	4.38E-02	±	1.27E-02
AdaFa- 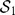	1.56E-55	±	4.84E-55	3.45E-28	±	1.05E-27	1.93E-54	±	5.11E-54	1.60E-28	±	2.95E-28
AdaFa- 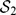	2.36E-87	±	9.56E-87	5.09E-45	±	8.84E-45	4.19E-86	±	9.73E-86	6.85E-45	±	1.68E-44
AdaFa- 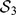	1.26E-123	±	3.17E-123	**7.44E-63**	±	**6.60E-63**	**2.16E-122**	±	**7.92E-122**	**1.30E-62**	±	**1.44E-62**
AdaFa- 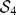	3.51E-78	±	1.15E-77	8.13E-40	±	2.33E-39	8.95E-77	±	3.37E-76	1.11E-39	±	2.06E-39
AdaFa- 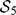	3.67E-74	±	4.93E-74	4.75E-38	±	3.59E-38	2.47E-73	±	4.25E-73	7.16E-38	±	6.57E-38
	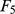			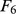			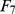			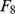		
SPSO	3.56E+01	±	2.84E+01	7.67E-01	±	1.01E+00	3.38E-03	±	1.36E-03	-7.28E+03	±	1.04E+03
APSO	2.94E+01	±	2.71E+01	6.67E-02	±	2.54E-01	1.17E-02	±	3.88E-03	-2.10E+04	±	2.96E+03
GPSO	3.92E+01	±	2.51E+01	6.67E-02	±	2.54E-01	1.27E-02	±	5.15E-03	**-1.30E+04**	±	1.13E+03
FA	8.28E+01	±	1.31E+02	**0.00E+00**	±	**0.00E+00**	3.50E-02	±	3.76E-02	-7.04E+03	±	6.91E+02
AdaFa- 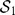	**2.87E+01**	±	1.82E-02	**0.00E+00**	±	**0.00E+00**	3.00E-05	±	3.05E-05	-4.83E+03	±	7.70E+02
AdaFa- 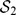	**2.87E+01**	±	**1.74E-02**	**0.00E+00**	±	**0.00E+00**	3.61E-05	±	4.76E-05	-4.87E+03	±	5.70E+02
AdaFa- 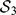	2.88E+01	±	2.08E-02	**0.00E+00**	±	**0.00E+00**	**2.20E-05**	±	**1.78E-05**	-4.96E+03	±	6.57E+02
AdaFa- 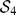	**2.87E+01**	±	2.01E-02	**0.00E+00**	±	**0.00E+00**	4.59E-05	±	4.18E-05	-4.90E+03	±	**5.40E+02**
AdaFa- 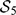	2.88E+01	±	2.26E-02	**0.00E+00**	±	**0.00E+00**	3.55E-05	±	2.96E-05	-4.94E+03	±	6.55E+02
	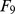			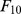			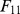			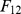		
SPSO	3.48E+01	±	3.03E+01	1.04E+00	±	8.29E-01	6.56E-03	±	1.12E-02	2.42E-02	±	8.47E-02
APSO	5.00E+01	±	1.37E+01	3.15E-01	±	5.88E-01	1.42E-02	±	1.73E-02	1.63E-01	±	2.34E-01
GPSO	3.30E+01	±	6.63E+00	8.41E-13	±	1.06E-12	1.05E-02	±	1.10E-02	3.11E-02	±	8.67E-02
FA	3.49E+01	±	1.31E+01	8.82E-03	±	1.32E-03	2.95E-03	±	2.24E-03	**5.87E-06**	±	**1.56E-06**
AdaFa- 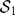	5.55E+00	±	3.04E+01	**4.56E-15**	±	**6.49E-16**	**0.00E+00**	±	**0.00E+00**	8.80E-02	±	1.69E-01
AdaFa- 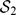	7.20E+00	±	2.74E+01	8.47E-15	±	5.00E-15	2.96E-17	±	9.64E-17	9.33E-02	±	2.06E-01
AdaFa- 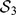	3.23E+00	±	1.77E+01	2.78E-14	±	2.09E-14	9.25E-16	±	1.31E-15	6.91E-03	±	2.63E-02
AdaFa- 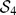	**3.03E-14**	±	**5.33E-14**	6.10E-15	±	1.80E-15	3.33E-17	±	8.82E-17	1.73E-02	±	5.50E-02
AdaFa- 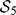	4.21E+00	±	2.30E+01	1.14E-14	±	3.55E-15	2.22E-17	±	6.12E-17	3.80E-02	±	1.07E-01

As illustrated in Table 1 and Fig. S1 in File S1, the five versions of AdaFa achieved better results than the other four algorithms on eight benchmark functions (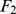
–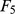, 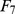, and 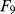
–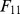), which can be also observed from Fig. S1 in File S1. SPSO achieved the target solution on function 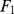, although the five versions of AdaFa did not as well as SPSO, they obtained the comparable results to each other, and the results were better than APSO, GPSO and FA. AdaFa-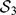 was better than AdaFa with other four different strategies on function 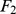
–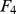, while other algorithms were all trapped and failed to obtain good results. It is easy to solve function 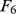 by the FA-based methods as shown in Table 1, as shown FA and five variants of AdaFa all found the target solutions to the problem. Due to the complex landscape of function 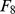, GPSO was the sole algorithm that found a most approaching results to the target. Similarly, AdaFa-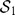 and AdaFa-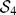 were the only ones that achieved sharp result than the compared algorithms on functions 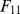 and 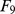, respectively. Although AdaFa-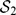, AdaFa-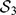 and AdaFa-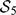 were all trapped into the local regions on function 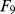, they performed so well that they all highly better than the three variants of PSO and FA on functions 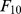 and 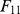. FA was good for the function 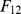, on which it was superior to others, but the rest possessed similar convergent characteristic as illustrated in Fig. S1 in File S1.

### Comparison of CPU efficiency

To compare the computational efficiency, we use CPU time to measure the complexity of each algorithm. For each algorithm, the computational efficiency is formulated as follows,
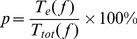
(21)where 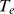 is the computational time of an algorithm on the benchmark function 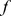, while the 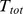 is the total time of all the algorithms on function 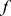.

**Figure 8:**
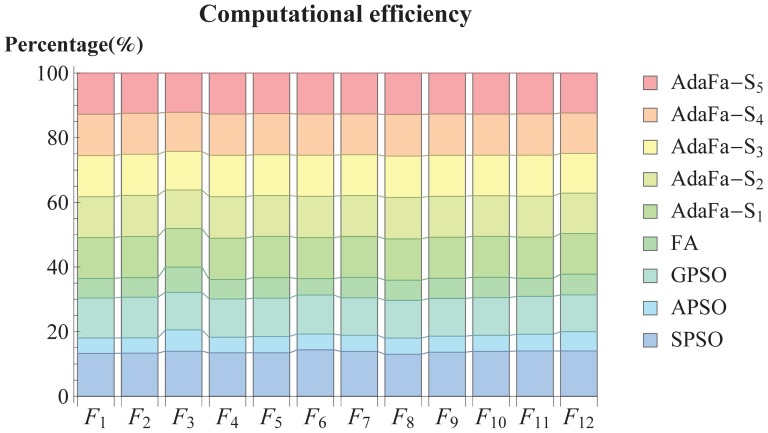
The comparison of computational efficiency of each algorithm on the test functions.

In Fig. 8, the computational efficiency is illustrated. As can be seen, APSO is the fastest among all the algorithms, and it is followed by FA. Their computational efficiency are quite similar to each other over each benchmark function. The costs of the proposed five versions of AdaFa are also similar to each other, and they are all comparable to those of SPSO. However, all the five version of AdaFa are worse than FA in terms of computational time due to several new mechanisms have been employed.

### Statistical analysis

Generally, it is necessary to use the non-parametric tests to analyze the experimental results. In this section, the Friedman, Aligned Friedman and Quade tests [22] were used to validate the performances of all the algorithms.

Table 2 presents the average rankings calculated from the Friedman, Aligned Friedman, and Quade tests. Each algorithm and its score are listed in ascending order. The statistics and the corresponding 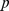-values are shown at the bottom of the table. In terms of computed 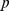-values, there exists significant differences among the algorithms with the 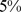 significance level.

**Table 2:** Average Ranks of all compared algorithms over all benchmark functions.

Average	Friedman		Aligned Friedman		Quade	
Rank	Algorithm	Score	Algorithm	Score	Algorithm	Score
1	AdaFa- 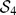	3.3333	AdaFa- 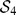	43.1667	AdaFa- 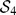	3.2756
2	AdaFa- 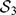	3.375	AdaFa- 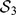	43.2083	AdaFa- 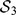	3.5833
3	AdaFa- 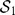	3.75	AdaFa- 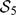	43.8333	AdaFa- 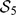	3.891
4	AdaFa- 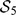	3.8333	AdaFa- 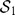	46	AdaFa- 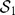	3.9167
5	AdaFa- 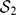	4.3333	AdaFa- 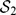	46.5833	AdaFa- 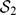	4.4551
6	GPSO	6.5	APSO	59.2917	APSO	6.109
7	APSO	6.5417	GPSO	66	GPSO	6.3462
8	FA	6.625	FA	69.4583	SPSO	6.7115
9	SPSO	6.7083	SPSO	72.9583	FA	6.7115
Statistic	30.35556		10.088976		3.022701	
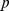 -value	0.000183		0.2588364		0.004785	

### Simulations on trans-membrane protein helix

Optimization approaches have been widely used in computational biology, for instance, immune algorithm (IA) was applied to discover a protein conformation with minimal energy based on lattice models [23]. Estimation of distribution algorithms (EDAs) [24] was used to solve the PSP in simplified models. Also based on simple protein models, Islam and Chetty employed memetic algorithm (MA) with several features to accomplish the structure prediction [25]. One important step of *ab initio* PSP methods is to reconstruct the tertiary structure of a protein by some optimization algorithms. Many methods reconstruct the tertiary structure of a protein depending on the residue contact maps, which can be solved in the framework of optimizing NP-hard problem [26], for example, in [27], a stochastic method based on simulated annealing (SA) was developed to derive a three-dimensional structure from a contact map. Vassura et al. used the a heuristic method and contact map to accelerate the process [28]. As we know, the aim of PSP is to obtain the Cartesian coordinates of all the atoms, which are bonded together by inter-atomic forces called chemical bonds. It has been observed that the bond lengths subject to a Gaussian distribution with a small standard deviation in high resolution protein structural data [29], and contain the essential information to determine the backbone structure of a protein [30]. Hence, given the torsion angle and bond length constrains, conformation of the geometry of the global protein structure is also an optimization problem, where the final structure resolution is significantly dependent on the algorithms.

In this study, we applied the developed AdaFa variants on the protein structure prediction problem with focus on the transmembrane helix proteins (TMHP). Membrane proteins account for 30% of the whole genome and more than 70% known drug targets. However, because of the hydrophobic environment of TMHP, they are the extreme difficult targets for the experimental structure biology studies [31], [32].

In high resolution protein structural data, it has been investigated that the bond lengths and angles subject to Gaussian distribution with a small standard deviation [33]. Here, we try to rebuild the tertiary structure directly from the bond lengths and angles with AdaFa. The data set of TMHP is filtered from the RCSB protein data bank (PDB) benchmark with 30% sequence identity cutoff, and it is released after Jan. 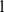
st, 2010. We use 30 TMHPs for the tests, where the number of the residues (Res. No.) is less than or equal to 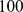, and the atomic resolutions of all the proteins are better than 2.5Å, without missed internal residues and sequence redundancies. These benchmark proteins are presented in Table 3.

**Table 3:** Information of Trans-membrane protein helix.

PDB ID	Res. No.	PDB ID	Res. No.	PDB ID	Res. No.
1afoA	40	2c0xA	50	2kdrA	28
1bzkA	42	2gofA	19	2l0eA	31
1eq8A	23	2h95A	18	2l6wA	39
1javA	19	2hacA	33	2lk9A	24
1lb0A	13	2htgA	27	2lx0A	32
1lcxA	13	2jtwA	25	2xkmA	46
1pjdA	15	2jwaA	44	3c9jA	25
1wazA	46	2k9jA	42	3e86A	70
1z65A	30	2k9yA	41	3hroA	37
2beqA	36	2ka1A	35	3mraA	25

From the 30 PDB structural data set, we extract only the angles of protein backbone including bond angles and torsion angles, which will be used as the constrains of the construction by the proposed AdaFa. In the experiments, the coordinate of the first backbone C_α_ atom was set randomly, the second one was determined by the standard bond length, the third one was fixed by two standard bond lengths and a bond angle, and the fourth one was calculated by the former three bond lengths, two bond angles and a dihedral angle. From the fifth C_α_ atom, the coordinates were determined by the same constrains involving four bond lengths, three bond angles and two dihedral angles. When considering the whole process as an optimization problem, we define the objective function as an energy, which is to calculate the distance between the undetermined C_α_ atom and its former four fixed C_α_ atom. For example, there are four determined coordinates of C_α_ atoms (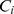, 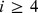), and we need to determine the coordinate of the 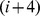
th C_α_ atom using the bond lengths, bond angles, and dihedral angles among the C_α_ atom 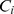, 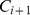, 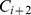 and 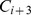 in the backbone of a chain. We transform the obtained knowledge into distance-based constrains in geometrical respect, and the distances can be denoted as 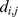, 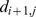, 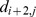 and 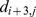 (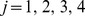). Accordingly, the energy can be formulated as 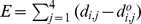, where 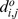 is transformed from the native constrains. The energy is used as the criterion of the proposed AdaFa in the optimization process. Hence, the backbone of a protein can be achieved by AdaFa iteratively. We then use the entire backbone of a protein chain as input of the PULCHRA [34] to determine the other atoms, such as hydrogen atoms and nitrogen atoms.

**Figure 9:**
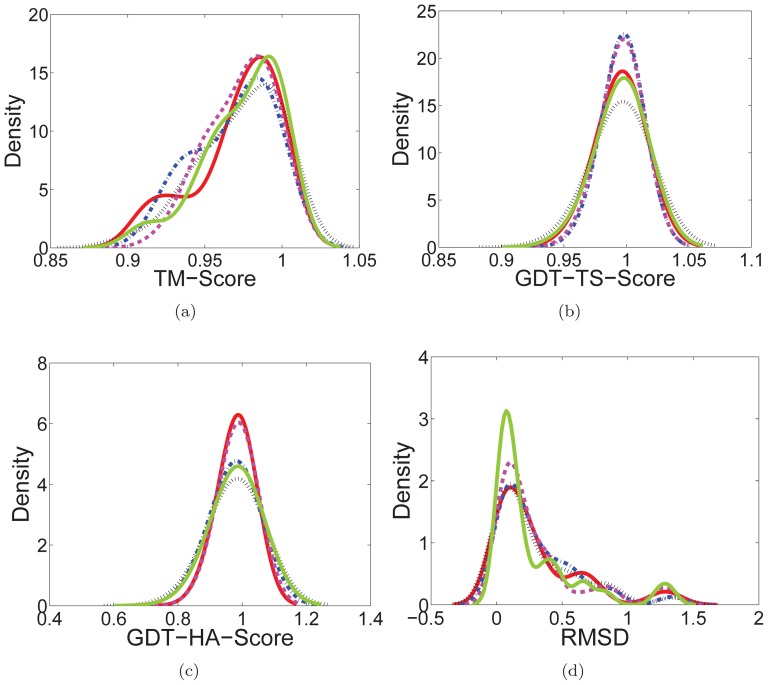
The kernel smoothing density estimate of (a) TM-Score, (b) GDT-TS-Score, (c) GDT-HA-Score, and (d) RMSD achieved over the native constrains. AdaFa-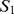
–AdaFa-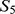 were represented by red solid line, black dotted line, blue dotted dashed line, magenta dashed line, and green solid line, respectively.

As illustrated in Fig. S2 in File S1, the tertiary structures of the proteins listed in Table 3 were predicted by AdaFa with high accuracy from the native constrains of each protein. In the experiments over 30 proteins, the averaged RMSDs of AdaFa-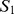
–AdaFa-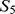 are 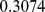
Å, 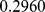
Å, 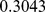
Å, 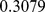
Å, and 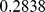
Å, respectively. To validate the robustness of AdaFa, the native constrains with 10% Gaussian white noise were used to predict the tertiary structures. According to the results in Fig. 9 and Fig. S3 in File S1, it can be seen that AdaFa variants (AdaFa-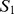
–AdaFa-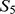) are able to construct the tertiary structure of the protein with high accuracy and good robustness, where the averaged RMSDs of AdaFa-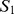
–AdaFa-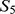 are 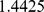
Å, 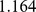
Å, 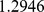
Å, 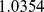
Å, and 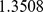
Å, respectively.

The different scores (TM-Score [35], GDT-TS-Score [36] and GDT-HA-Score [37]) and RMSD are illustrated by kernel smoothing density estimate [38] in Fig. 9 (More details can be found in Supporting Information). From Fig. 9 (a), the proposed AdaFa with five various strategies achieved different TM-Scores, in which AdaFa-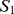, AdaFa-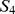 and AdaFa-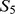 were better than the rest two variants of AdaFa. AdaFa-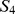 was superior to the other one in terms of GDT-TS-Score and GDT-HA-Score as shown in Fig. 9 (b) and Fig. 9 (c), while the AdaFa-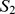 was the worst one among the variants of AdaFa over the protein benchmark dataset. On the other hand, AdaFa-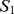 and AdaFa-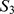 exhibited a little different and competed with each other in the two score items. In respect of RMSD as shown in Fig. 9 (d), AdaFa-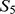 occupied an overwhelming position, which was far better than the other four AdaFa variants, among which AdaFa-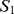
–AdaFa-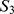 possessed similar density estimation.

## Conclusion

In this paper, we develop an adaptive firefly algorithm (AdaFa) and its five variants to enhance the search ability of the original FA. In AdaFa, we propose a distance-based technique to overcome the two main drawbacks in using a constant light absorption coefficient, which tunes the light among the fireflies dynamically to control the sharing distance information leading to the variation of the attractiveness. Simultaneously, the differences among the fireflies can be adequately used to enhance the local search ability of each firefly, hence we employ the gray relational analysis to design a gray coefficient as another self-adaptively altering parameter. According to the designed parameters, AdaFa uses heterogeneous update laws to accomplish the balance between the exploitation and the exploration throughout the search process.

In the numerical experiments, we compared the performances of all five proposed AdaFa variants with FA and other three PSO-based algorithms, and the statistical results demonstrated the five AdaFa variants were significantly better than the other four algorithms with the 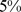 significance level. The experiments on the reconstruction of the tertiary structure of the protein showed that AdaFa had the potential ability in predicting the helix structures with high accuracy and good robustness. Although AdaFa exhibited good performance on either the numerical experiments or real-world application on the prediction of the proteins' tertiary structures, it is still a challenging problem to deal with the cooperation among the fireflies for further improving the performance of FA, which is our future efforts. The codes of AdaFa is available upon request at http://www.csbio.sjtu.edu.cn/bioinf/AdaFa-PAA/.

## Supporting Information

Algorithm S1 **The FA algorithm.**
(TXT)

Algorithm S2 **The AdaFa algorithm.**
(TXT)

File S1 **Combined file of supporting figures and tables.** Figure S1: The mean value over the benchmark functions with 30-dimensions. Figure S2: Simulation results over thirty proteins. Figure S3: The kernel smoothing density estimates of different measurement metrics. Table S1: Benchmark Functions.
(ZIP)
