# Supplementary material for: Adaptive Firefly Algorithm: Parameter Analysis and its Application
Source: PLoS One. 2014 Nov 14;9(11):e112634. doi: 10.1371/journal.pone.0112634 (PMC4232507; doi:10.1371/journal.pone.0112634)
Supplement: File S1 — Combined file of supporting figures and tables. Figure S1: The mean value over the benchmark functions with 30-dimensions. Figure S2: Simulation results over thirty proteins. Figure S3: The kernel smoothing density estimates of different measurement metrics. Table S1: Benchmark Functions. (ZIP) [file pone.0112634.s003.zip › si/AdaFa - PAA - SI.pdf]

Supplementary Materials
Adaptive Firefly Algorithm: Parameter Analysis and its Application

1 Numerical experiments

Table S1: Benchmark Functions

	Test function	Search range	f_{min}
Unimodal	$F_1(X) = \sum_{i=1}^{D} x_i^2$	[-100,100]^D	0
	$F_2(X) = \sum_{i=1}^{D} \lvert x_i \rvert + \prod_{i=1}^{D} \lvert x_i \rvert$	[-10,10]^D	0
	$F_3(X) = \sum_{i=1}^{D} \left(\sum_{j=1}^{i} x_j \right)^2$	[-100,100]^D	0
	$F_4(X) = \sum_{i=1}^{D-1} \left[100(x_i^2 - x_{i+1})^2 + (x_i - 1)^2 \right]$	[-10,10]^D	0
	$F_5(X) = \sum_{i=1}^{D} \lfloor x_i + 0.5 \rfloor^2$	[-100,100]^D	0
	$F_6(X) = \sum_{i=1}^{D} i x_i^4 + random[0,1)$	[-1.28,1.28]^D	0
Multimodal	$F_7(X) = \sum_{i=1}^{D} \left(-x_i \sin(\sqrt{\lvert x_i \rvert}) \right)$	[-500,500]^D	-418.9829×D
	$F_8(X) = \sum_{i=1}^{D} \left(x_i^2 - 10\cos(2\pi x_i) + 10 \right)$	[-5.12,5.12]^D	0
	$F_9(X) = \sum_{i=1}^{D} \left(y_i^2 - 10\cos(2\pi y_i) + 10 \right)$ where $y_i = \begin{cases} x_i & \lvert x_i \rvert < 0.5 \\ \frac{round(2x_i)}{2} & \lvert x_i \rvert \geqslant 0.5 \end{cases}$	[-5.12,5.12]^D	0
	$F_{10}(X) = -20exp\left(-0.2\sqrt{\frac{1}{D}\sum_{i=1}^{D} x_i^2} \right) - exp\left(\frac{1}{D}\sum_{i=1}^{D} \cos(2\pi x_i) \right) + 20 + e$	[-32,32]^D	0
	$F_{11}(X) = \frac{1}{4000}\sum_{i=1}^{D} x_i^2 - \prod_{i=1}^{D} \cos(\frac{x_i}{\sqrt{i}}) + 1$	[-600,600]^D	0
	$F_{12}(X) = \frac{\pi}{D}\left\{ 10\sin^2(\pi y_i) + \sum_{i=1}^{D-1}(y_i - 1)^2[1 + 10\sin^2(\pi y_{i+1})] + (y_D - 1)^2 \right\} + \sum_{i=1}^{D} u(x_i, 10, 100, 4)$ where $y_i = 0.25(x_i + 1) + 1$, $u(x_i, a, k, m) = \begin{cases} k(x_i - a)^m & x_i \geqslant a \\ 0 & -a < x_i < a \\ k(-x_i - a)^m & x_i < -a \end{cases}$	[-50,50]^D	0

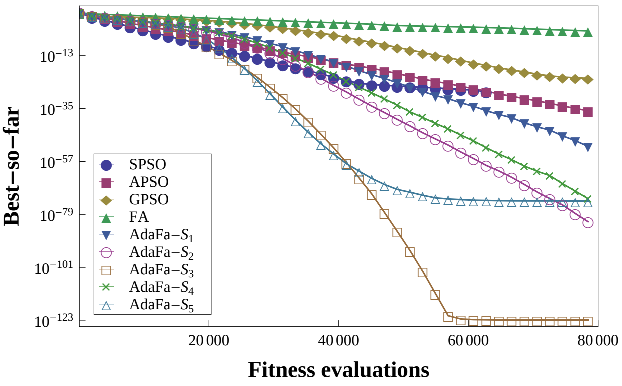

(a) F_1

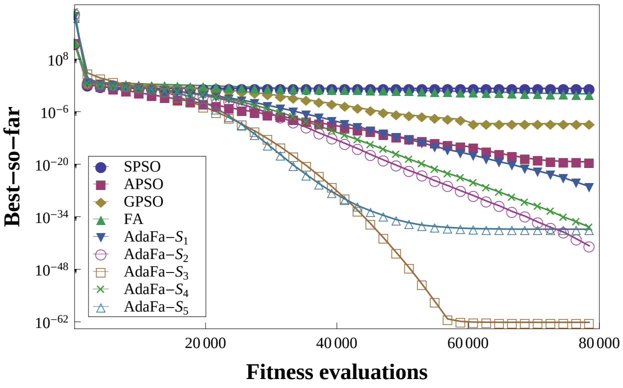

(b) F_2

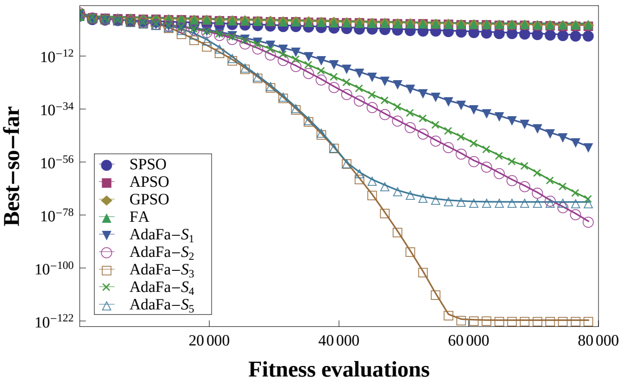

(c) F_3

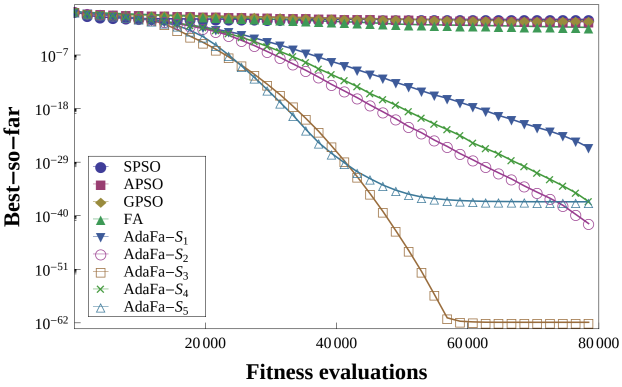

(d) F_4

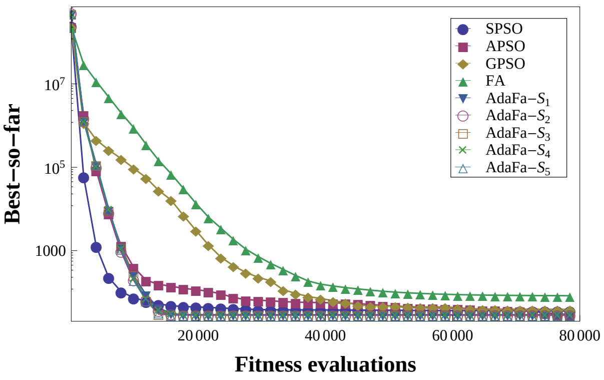

(e) F_5

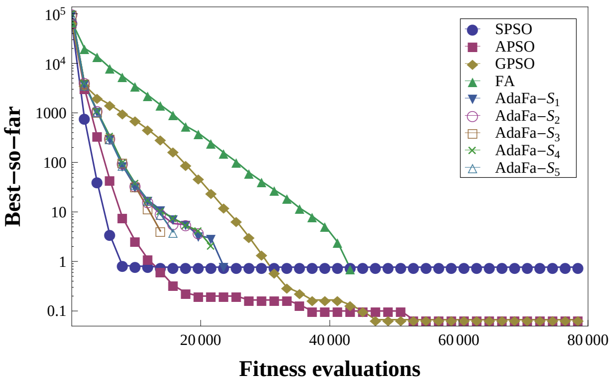

(f) F_6

Fig. S1: The mean value over the benchmark functions with 30-dimensions.

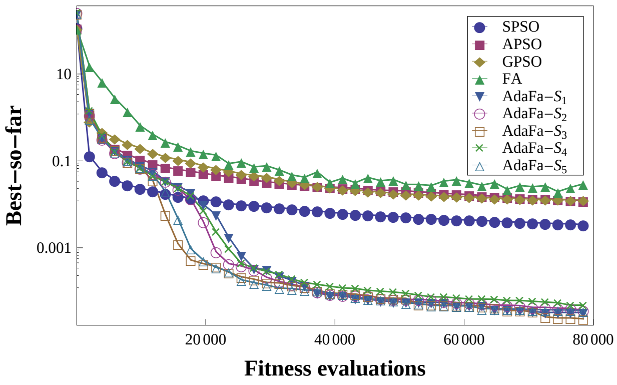

(a) F_7

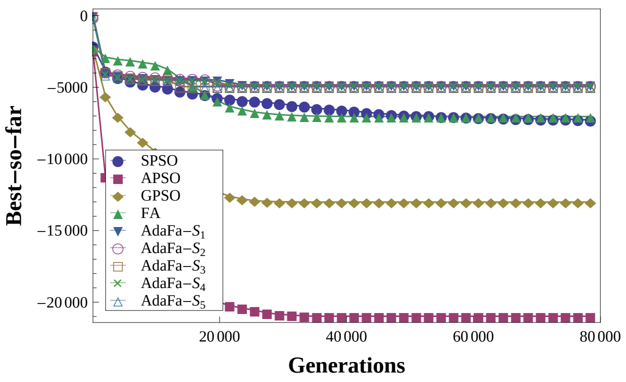

(b) F_8

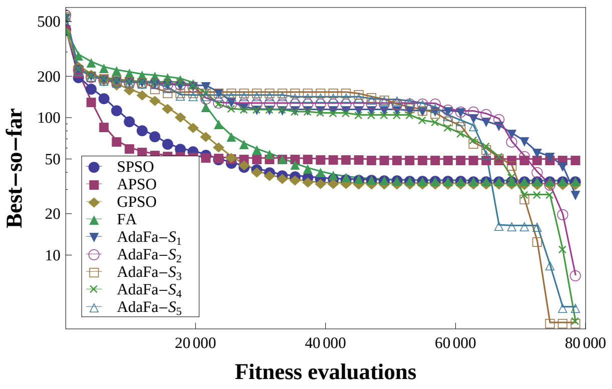

(c) F_9

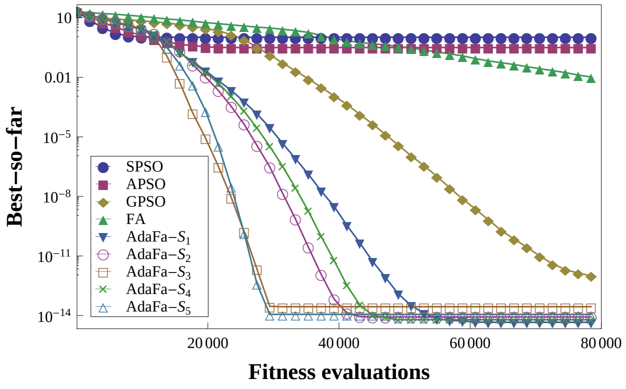

(d) F_{10}

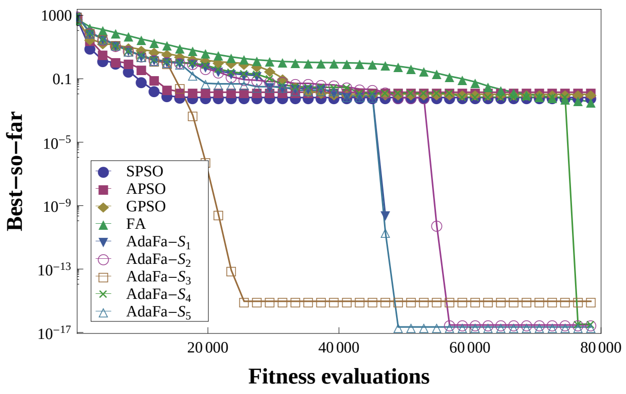

(e) F_{11}

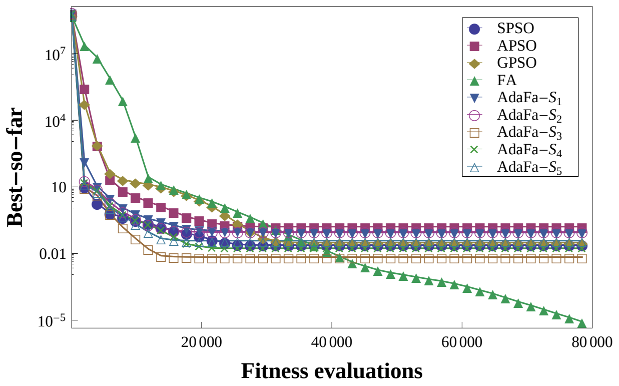

(f) F_{12}

Fig. S1: (*Continue.*) The mean values over the benchmark functions with 30-dimensions.

2 Applications of AdaFa variants

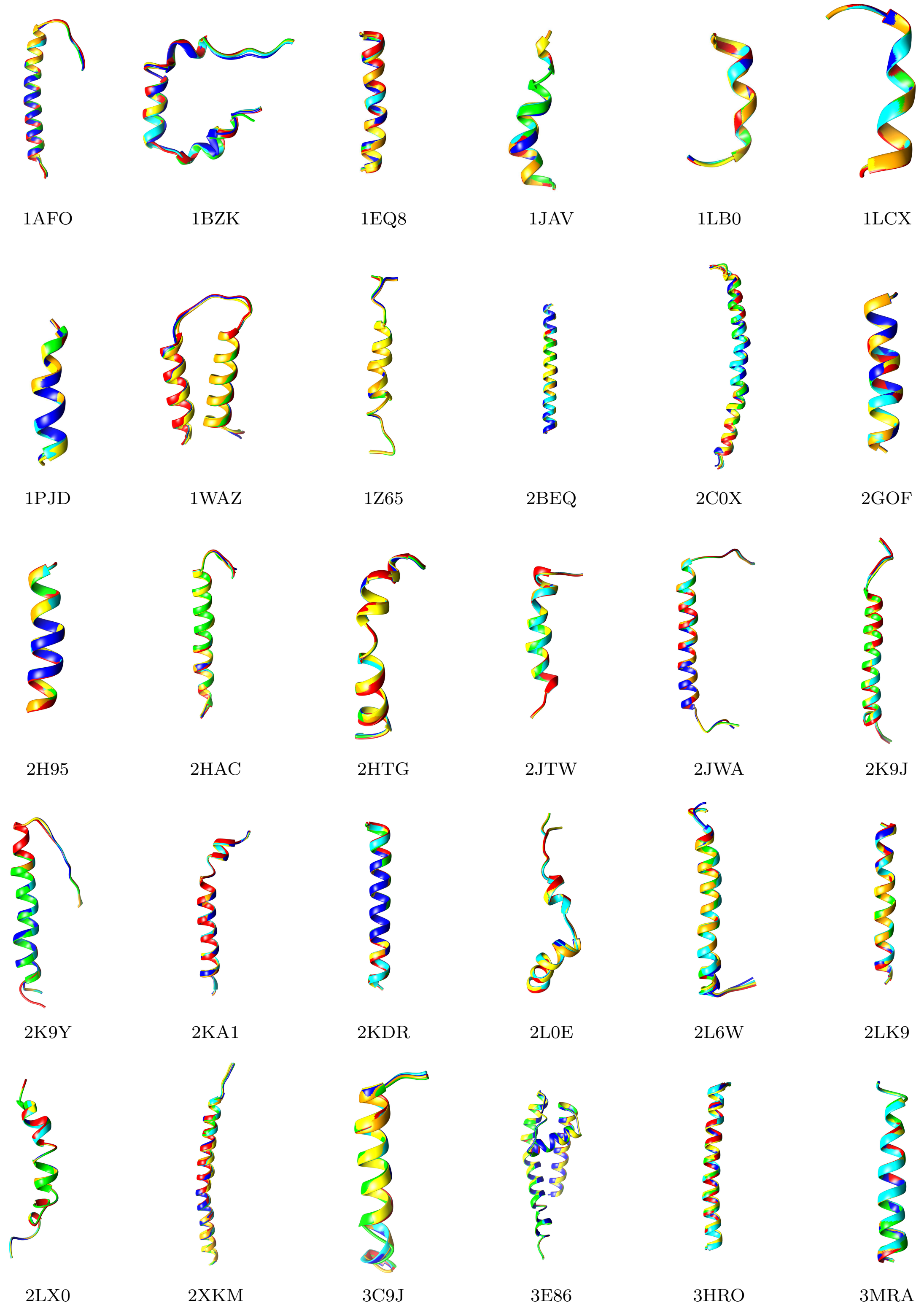

Fig. S2: The similarity between native protein structure (red) and the reconstructed structures (AdaFa-S_1: blue, AdaFa-S_2: green, AdaFa-S_3: yellow, AdaFa-S_4: cyan, AdaFa-S_5: orange).

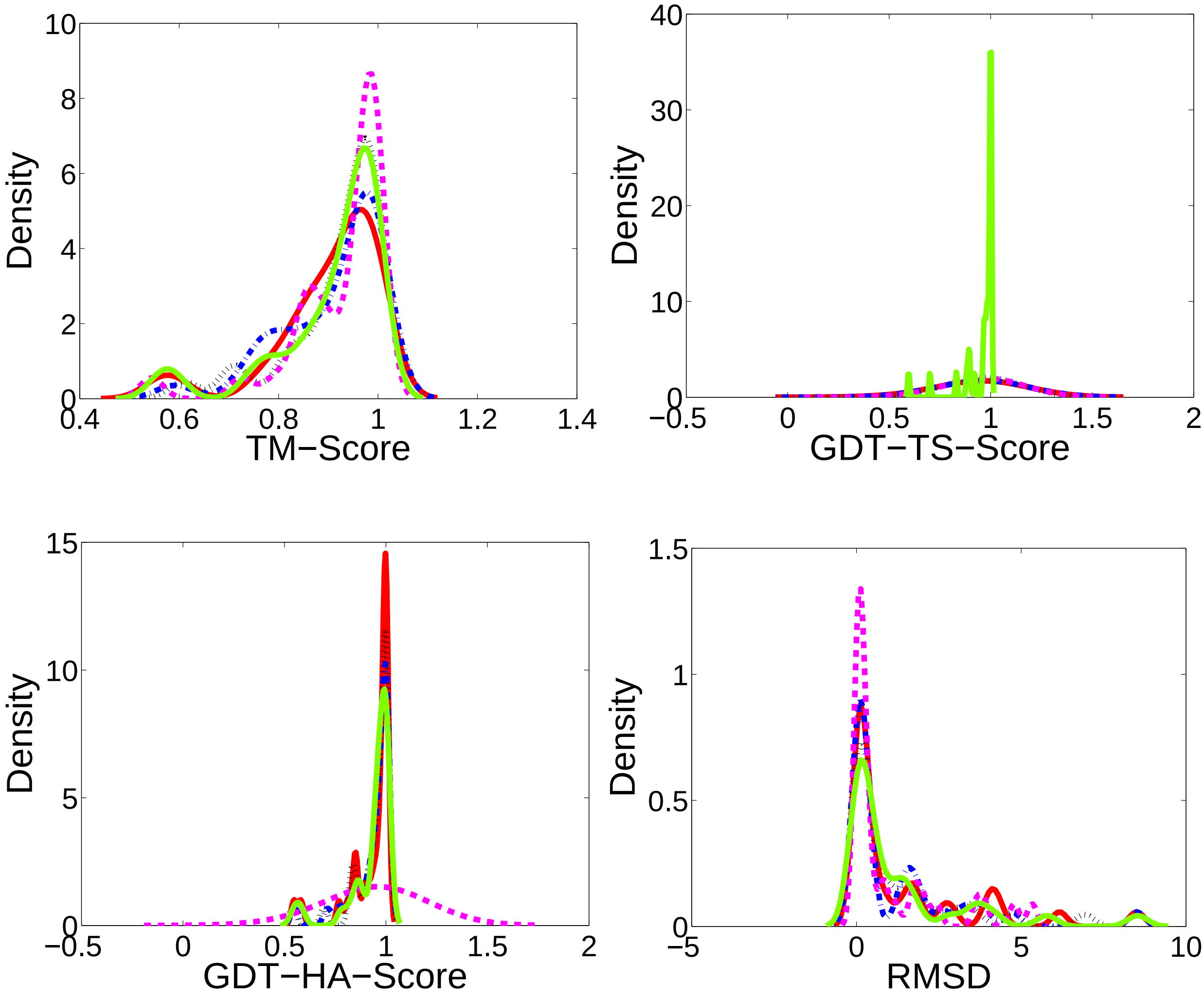

Fig. S3: The kernel smoothing density estimates of (a) TM-Score, (b) GDT-TS-Score, (c) GDT-HA-Score, and (d) RMSD achieved over the constrains with 10% Gaussian white noise. AdaFa-S_1–AdaFa-S_5 were represented by red solid line, black dotted line, blue dotted dashed line, magenta dashed line, and green solid line, respectively
