# Supplementary figures and images for: Adaptive Firefly Algorithm: Parameter Analysis and its Application
Source: PLoS One. 2014 Nov 14;9(11):e112634. doi: 10.1371/journal.pone.0112634 (PMC4232507; doi:10.1371/journal.pone.0112634)

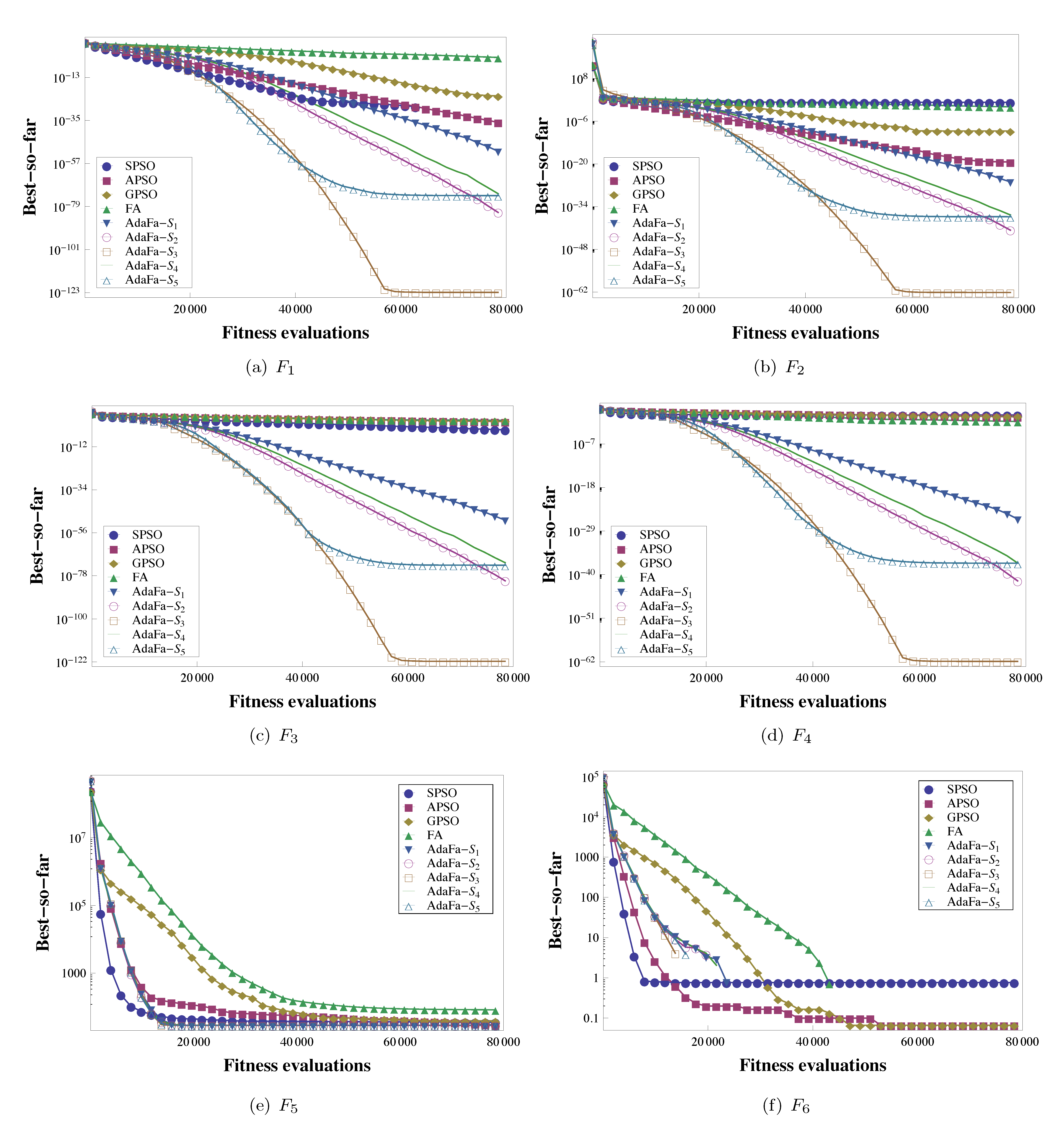

Supplement: File S1 — Combined file of supporting figures and tables. Figure S1: The mean value over the benchmark functions with 30-dimensions. Figure S2: Simulation results over thirty proteins. Figure S3: The kernel smoothing density estimates of different measurement metrics. Table S1: Benchmark Functions. (ZIP) [file pone.0112634.s003.zip › si/Figure S1-1.tiff]

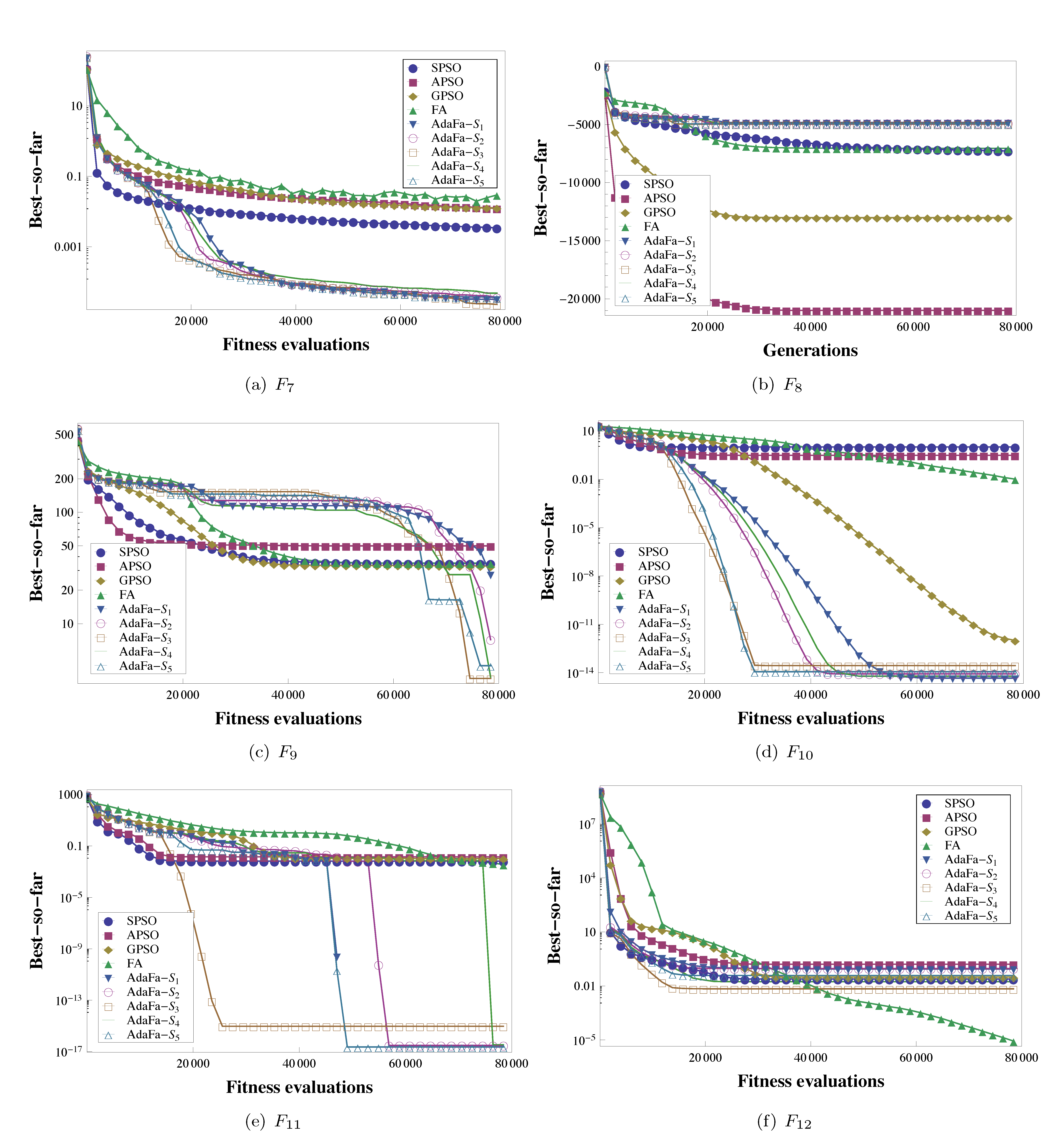

Supplement: File S1 — Combined file of supporting figures and tables. Figure S1: The mean value over the benchmark functions with 30-dimensions. Figure S2: Simulation results over thirty proteins. Figure S3: The kernel smoothing density estimates of different measurement metrics. Table S1: Benchmark Functions. (ZIP) [file pone.0112634.s003.zip › si/Figure S1-2.tiff]

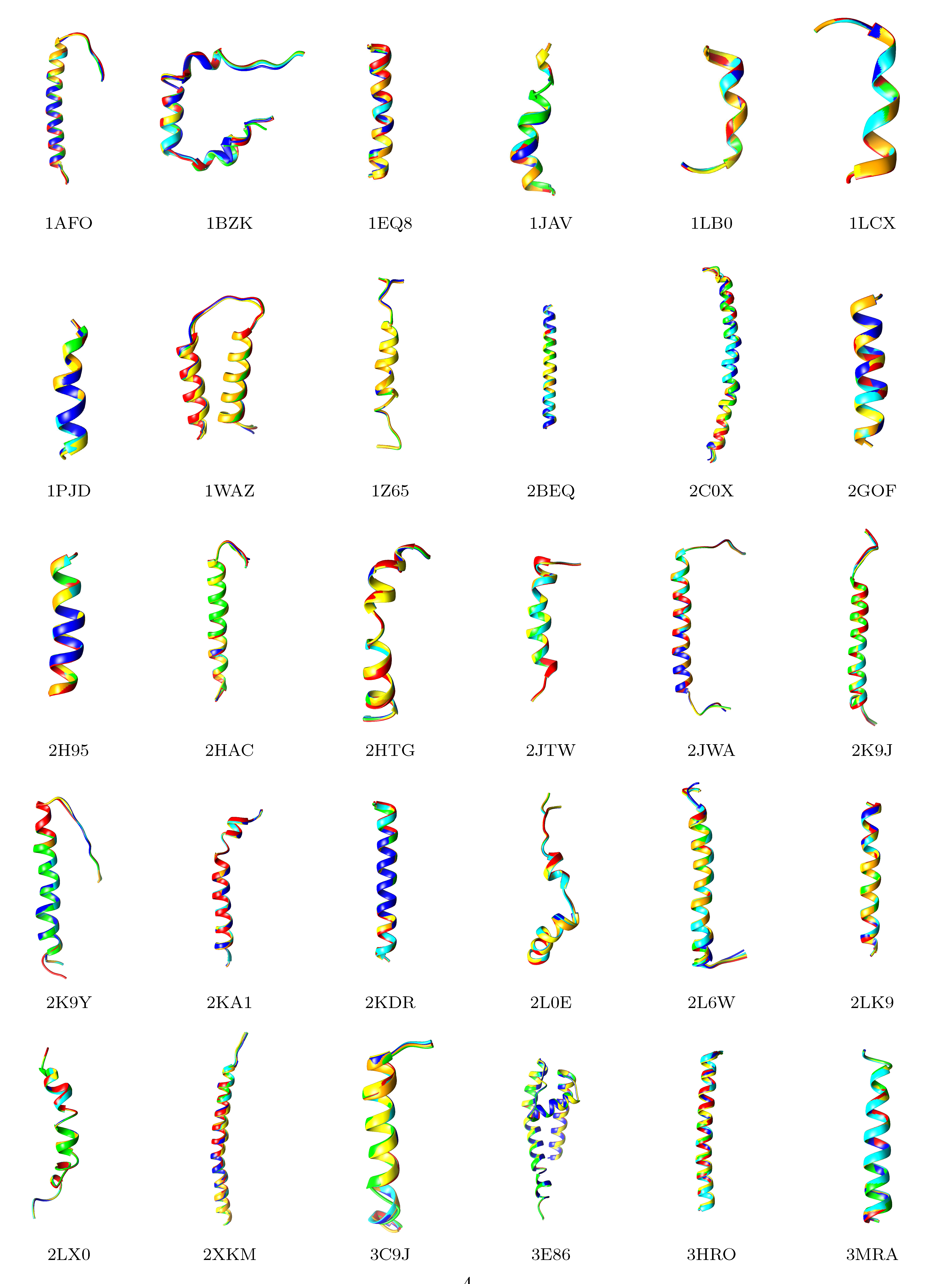

Supplement: File S1 — Combined file of supporting figures and tables. Figure S1: The mean value over the benchmark functions with 30-dimensions. Figure S2: Simulation results over thirty proteins. Figure S3: The kernel smoothing density estimates of different measurement metrics. Table S1: Benchmark Functions. (ZIP) [file pone.0112634.s003.zip › si/Figure S2.tiff]

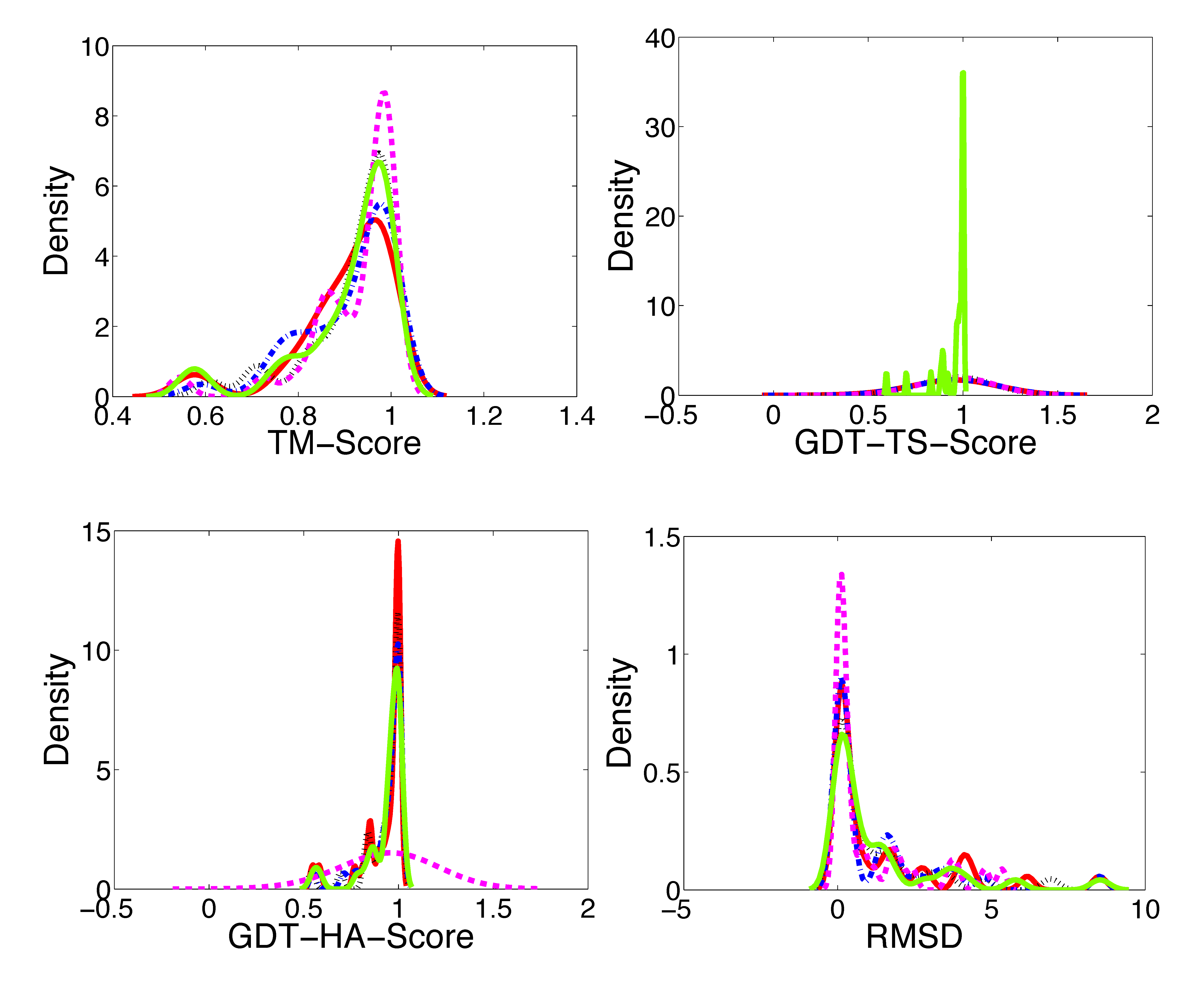

Supplement: File S1 — Combined file of supporting figures and tables. Figure S1: The mean value over the benchmark functions with 30-dimensions. Figure S2: Simulation results over thirty proteins. Figure S3: The kernel smoothing density estimates of different measurement metrics. Table S1: Benchmark Functions. (ZIP) [file pone.0112634.s003.zip › si/Figure S3.tiff]
